# DNA lesions and repair in trypanosomatids infection

**DOI:** 10.1590/1678-4685-GMB-2019-0163

**Published:** 2020-03-27

**Authors:** Bruno M. Repolês, Carlos Renato Machado, Pilar T.V. Florentino

**Affiliations:** 1 Universidade Federal de Minas Gerais, Departamento de Bioquímica e Imunologia, Belo Horizonte MG, Brazil.; 2 Universidade de São Paulo, Departamento de Microbiologia, São Paulo, SP, Brazil.

**Keywords:** Trypanosoma cruzi, reactive oxygen species, DNA repair

## Abstract

Pathological processes such as bacterial, viral and parasitic infections can generate a plethora of responses such as, but not restricted to, oxidative stress that can be harmful to the host and the pathogen. This stress occurs when there is an imbalance between reactive oxygen species produced and antioxidant factors produced in response to the infection. This imbalance can lead to DNA lesions in both infected cells as well as in the pathogen. The effects of the host response on the parasite lead to several kinds of DNA damage, causing alterations in the parasite’s metabolism; the reaction and sensitivity of the parasite to these responses are related to the DNA metabolism and life cycle of each parasite. The present review will discuss the survival strategies developed by host cells and *Trypanosoma cruzi,* focusing on the DNA repair mechanisms of these organisms throughout infection including the relationship between DNA damage, stress response features, and the unique characteristics of these diseases.

## Introduction

The Kinetoplastida order comprises several Protozoa that can be parasitic or not. They share some unique characteristics, most remarkably the presence of a single and unique organelle called a kinetoplast. In this order, three of the major parasitic pathogens of medical importance are part of the Trypanosomatid family: *Leishmania spp*. (the etiological agent of the various forms of leishmaniasis), *Trypanosoma brucei* (the causative agent of African sleep sickness) and *Trypanosoma cruzi* (the etiological agent of Chagas disease, also named American trypanosomiasis). All these organisms have a heteroxenous life cycle, needing two hosts one invertebrate and another vertebrate; therefore, they present more than one parasitic form (Chagas, 1909; Rassi *et al.*, 2010; Cantey *et al.*, 2012; Centers for Disease and Control Prevention, 2018). Generally, diseases caused by trypanosomatids are zoonotic, i.e., they have animals as a natural reservoir and infection in humans is dependent on the maintenance of these reservoirs to sustain endemicity. Although *T. cru*zi and *T. brucei* are in the same genus, they have a distinct life cycle. Information about the *T. cruzi* life cycle is summarized in Box 1.

Box 1 ***Trypanosoma cruzi* life cycle**

*Trypanosoma cruzi* is a heteroxenous parasite with a digenetic life cycle that involves two hosts: an invertebrate one, usually an insect of the order Triatomina and a vertebrate host. In each of these hosts and stages of development, the parasite assumes a different morphology. *T. cruzi* infection can occur by transfusion using contaminated blood, oral infection via contaminated food, or by direct infection by the insect vector. In a typical *T. cruzi* life cycle, the trypomastigote form of the parasite is deposited in the feces of the insect vector. The infection occurs when the feces come into contact with any mucosal tissue or the bloodstream. After that, the parasite is able to infect several types of cells, including macrophages, cardiomiocytes, adipocytes, epithelial cells, and neurons. During infection, *T. cruzi* trypomastigotes form a parasitophorous vacuole inside the host cell, and then escape from this vacuole into the cytoplasm. The parasite then differentiates into the replicative amastigote cell, further replicates, and transforms again into the trypomastigote form. Finally, the host cell bursts and releases new parasites to continue the infection cycle. When another insect vector feeds on blood containing trypomastigotes parasites, *T. cruzi* then reaches the intestinal tract of this insect. In the insect gut, the parasite differentiates into the replicative epimastigote form, replicates again, then differentiates into the trypomastigote, which then undergoes another infection cycle within the mammalian host.

Several reviews have already addressed the particularities of how the trypanosomatids escape from the immune system (Oladiran and Belosevic, 2012; Geiger *et al.*, 2016; Weatherly *et al.*, 2016). Although *T. brucei* has recently been reported in the adipocytes of mice (Trindade *et al.*, 2016), this parasite is present in a bloodstream-specific form in mammals and, therefore, has to deal with the attack of the host’s immune system. To be able to escape from the immune system, *T. brucei* has an extensive repertoire of variant surface glycoproteins (VSGs) that must be constantly exchanged (Taylor and Rudenko, 2006); this indicates a role for homologous recombination in the DNA metabolism of this parasite (McCulloch and Barry, 1999; Glover *et al.*, 2008; Horn and McCulloch, 2010).

For *T. cruzi,* the first barrier is immune system recognition in the bloodstream. In the mammalian host, bloodstream *T. cruzi* has to combat the host’s complement system, and therefore, the parasite expresses several proteins that interfere with the complement system. One of those proteins is T-DAF (trypomastigote decay-accelerating-factor), a protein capable of blocking the assembly of the C3 convertases from the complement system. The parasite also expresses several complement regulatory proteins (CRPs) and calreticulin (CRT) that also interfere with the complement system (Norris *et al.*, 1991; Tambourgi *et al.*, 1993; Cestari *et al.*, 2009; Ramírez *et al.*, 2011). The parasite also possesses trans-sialidases proteins that interfere with host lymphocytes as well as sialylated mucins and cruzipain that actively protect the parasite against antibodies from the host immune system (Berasain *et al.*, 2003; Giorgi and De Lederkremer, 2011; Alvarez *et al.*, 2012). The intracellular amastigote form requires an extensive repertoire of oxidative response proteins to cope with the new intracellular environment. During the intracellular phase, the host cell activates several oxidases in the parasitophorous vacuole, creating an oxidative burst via the generation of several reactive oxygen species (ROS) that can attack the parasite. The parasite’s response repertoire must also include DNA damage response proteins to oxidative stress (Passos-Silva *et al.*, 2010; van Loon *et al.*, 2010; Alvarez *et al.*, 2011; Genois *et al.*, 2014; Machado-Silva *et al.*, 2016).

During Chagas disease, *T. cruzi* has an intracellular form that must also resist the oxidative insult caused by the host cell. The primary response against the parasite is the oxidative burst caused during the infection, which consists mainly of superoxide anions and ROS (Müller *et al.*, 2003; Alvarez *et al.*, 2004; Piacenza *et al.*, 2009a,b). This insult is the most deleterious for the parasite and, therefore, the most studied. These pathological processes that generate oxidative stress can be very harmful to the individual and pathogen (Nathan and Shiloh, 2002). Stress occurs when there is an unbalance between reactive oxygen species (ROS: hydroxyl radical, nitric oxide, superoxide, hydrogen peroxide, hypochlorous acid, and singlet oxygen) and antioxidant species (superoxide dismutase, glutathione reductase, glutathione, α-tocopherol, and ascorbic acid). The main reactive oxygen species (ROS) targets include DNA, RNA, lipids, proteins, and carbohydrates. However, DNA lesions result in cell cycle arrest and death, as it is the molecule responsible for genetic information of all cells from a single organism (Berra *et al.*, 2006). Beyond oxidative stress, the importance of preserving genomic integrity is evident by the fact that DNA repair mechanisms are present in all organisms, as we have the description of DNA repair proteins and enzymes from bacteria to higher eukaryotes (Hoeijmakers, 2001; DiRuggiero and Robb, 2004; Cagney *et al.*, 2006).

During the cell cycle, DNA repair pathways ensure the fidelity of the DNA information transferred (Friedberg, 2008). The complexity of these processes becomes clear when we consider that, for any eukaryotic cell, there are numerous sources of DNA damage, which need to be detected and repaired (Hoeijmakers, 2001; Hakem, 2008; Tubbs and Nussenzweig, 2017). DNA damage response leads to different cellular effects, such as cell cycle arrest, activation of distinct signaling pathways, and modulation of the DNA metabolism pathways (Hoeijmakers, 2001; Marnett and Plastaras, 2001; Harper and Elledge, 2007; Lord and Ashworth, 2012; Imlay, 2013). Therefore, although the DNA damage response is divided into pathways, the alterations in the cell comprise other metabolic pathways that are not so logical at first glance.

In this review, we will discuss what is already known about DNA metabolism and repair during *T. cruzi* infection and how these processes affect host cell metabolism. Thus, we will explore how DNA metabolism may be related to alterations in cellular metabolism and how these changes impact the pathogenesis of Chagas disease.

## Metabolic effects of oxidative stress in *T. cruzi*

As an obligate intracellular parasite, *T. cruzi* has to combat oxidative stress generated by the host cell. The primary host response against the parasite is the oxidative burst caused by the infection, which consists mainly of superoxide anions and ROS (Müller *et al.*, 2003; Alvarez *et al.*, 2004; Piacenza *et al.*, 2009a,b).

With such specific environmental insults during the parasite life cycle, *T. cruzi* reacts with several metabolic responses to attempt to prevent the damage caused by ROS. A significant protein in this response is a parasite-specific superoxide dismutase (SOD), a protein already reported to protect the parasite from intracellular-generated ROS by macrophages (Mateo *et al.*, 2008; Martinez *et al.*, 2018). SOD is an enzyme that catalyzes the dismutation of superoxide (O_2_^-^) into hydrogen peroxide (H_2_O_2_). In *T. cruzi* the SOD enzyme does not possess a copper/zinc or manganese mechanism; *T. cruzi* SODs are present in four iron-dependent versions of SOD (FeSOD) A, B1, B2, and C. It has been demonstrated that the overexpression of FeSOD C within the mitochondria was able to improve the resistance of the pathogen to ROS generated by the presence of bovine serum (Boveris and Stoppani, 1977; Piacenza *et al.*, 2007).

Contrary to most organisms, trypanosomatid glutathione is a tiny fraction of the pool of enzymes. Most eukaryotes rely on a system composed of glutathione and glutathione reductase in a system that uses glutathione as reductive power to neutralize oxidative molecules, in a reaction catalyzed by glutathione reductase (Krauth-Siegel *et al.*, 2003; Müller *et al.*, 2003). In these parasites, the trypanothione system is responsible for protecting those organisms from oxidative insult (Fairlamb *et al.*, 1985; Fairlamb and Cerami, 1992; Meziane-Cherif *et al.*, 1994). This protein is homologous to the human glutathione reductase, an enzyme capable of performing the reduction of glutathione disulfide into the sulfhydryl form glutathione (Fairlamb *et al.*, 1985; Piacenza *et al.*, 2009b). Although this protein is essential to the response of *T. cruzi* to oxidative assault, there is still some debate regarding its subcellular localization in the parasite, as the presence of trypanothione reductase was reported in the parasite’s kinetoplast (Meziane-Cherif *et al.*, 1994), yet fractionation studies showed that this localization might be more diffuse than previously thought (Wilkinson *et al.*, 2002).

One of the main classes of proteins involved in the cytosolic response against ROS in *T. cruzi* is the family of peroxiredoxins. Two of these well-characterized proteins in *T. cruzi* are the mitochondrial and cytosolic tryparedoxin perioxidase proteins (TcMPx and TcCPx, respectively) (Piacenza *et al.*, 2009b). Overexpression of these proteins can confer resistance to peroxynitrite. Expression of both proteins differs among the *T. cruzi* forms, as it is higher in metacyclic trypomastigotes than in epimastigotes (Piacenza *et al.*, 2009b). The existence of several proteins to deal with oxidative stress indicates how important the oxidative response is for the parasite.

## DNA repair and response to oxidative stress in *T. cruzi*

The oxidative stress that surpasses the first line of defense of the parasite, composed by the aforementioned antioxidant proteins, constitutes a potential source of damage to the intracellular components of the organism. One primary target of ROS in living organisms is DNA. Among many effects, oxidative stress can generate apurinic/apyrimidinic (AP) sites and base modifications, one of the most common of which is the modified base 7,8-dihydro-8-oxoguanine also known as 8-oxoguanine (8-oxodG) (Kanvah *et al.*, 2010). Further studies of the response of *T. cruzi* to the damage caused by 8-oxoguanine in each forms of the parasite are necessary since one of the two crucial drugs used against this parasite, benznidazole (BZN), is able to induce oxidative stress primarily by targeting guanine in the nucleotide pool of the parasite (Rajão *et al.*, 2014).

8-oxoguanine can be generated directly on double-stranded DNA, but also by the oxidation of the guanine present on the cell nucleotide pool (Mahon *et al.*, 2007; Aguiar *et al.*, 2013). This modified base mutagenicity is due to its ability to cause transversions when not corrected by the DNA repair system since replicative polymerases can incorporate cytosine or adenine in opposition to 8-oxodG. Therefore, this modified base can be incorporated in opposition to an adenine, causing the transversion AT to CG (Cadet *et al.*, 2003; Barzilai and Yamamoto, 2004; van Loon *et al.*, 2010).

There are several pieces of evidence indicating that DNA repair factors are not restricted to one pathway, but instead can be involved with the regulation and control of multiple DNA repair pathways (Nemzow *et al.*, 2015). Although the response against oxidative insult is very complex and involves multiple metabolic alterations (Pascucci *et al.*, 2011; Melis *et al.*, 2013), the primary pathway involved in DNA repair is base excision repair (BER) (Hoeijmakers, 2001; Barzilai and Yamamoto, 2004). In general terms, the BER pathway consists of several DNA glycosylases, each one specifically recognizing one type of modified base. These proteins can flip the modified base out of the DNA strand and cleave it from the sugar-phosphate backbone. This cleavage will generate an AP site, cleaned by some endonucleases; sometimes this hydrolysis occurs spontaneously (Meadows *et al.*, 2003). From this point forward the pathway can take two directions: the short-patch BER, in which a polymerase, such as polymerase β (Polβ), performs a single nucleotide gap fill on the damage site, or the long-patch BER, in which, in coordination with proliferating cell nuclear antigen (PCNA), polymerization of a more substantial portion of the DNA and displacement of the damaged single-strand occurs, generating a DNA flap. Next, the DNA flap is removed by flap endonuclease 1 (FEN1), and the DNA nick is closed by a specific DNA ligase (Hoeijmakers, 2001; David *et al.*, 2010; Wallace 2014).

The oxidative response is one of the main fields of study for *T. cruzi* and proteins involved in all steps of both BER pathways have already been described (Passos-Silva *et al.*, 2010; Genois *et al.*, 2014). One significant protein already reported in *T. cruzi* is the homolog for AP endonuclease, the enzyme responsible for cleaning AP sites from the genome, thereby preventing cytotoxic effects during replication. Two AP endonuclease (APE) homologs were reported: TcAPE1 (orthologous to *Homo sapiens* APE1) and TcAP2 (orthologous to *Homo sapiens* APE 2 and *Schizosaccharomyces pombe* Apn2p) (Sepúlveda *et al.*, 2014). These proteins were identified in replicative forms of the parasite, demonstrating the importance of their expression for accurate DNA replication. The overexpression of these proteins confers higher resistance to oxidative stress, especially in a continuous and persistent oxidative environment, when compared with wildtype (WT) strains, thus demonstrating how essential DNA repair is to the survival of the parasite in the face of oxidative attacks (Sepúlveda *et al.*, 2014). Although there is much investigation into the epimastigote form, there is lack information regarding the influence of these genes in the amastigote form of the parasite.

One piece of evidence for the presence of the short-patch BER in *T. cruzi* is the observation that the parasite can cope with uracil by using the protein Tc-uracil DNA glycosylase (TcUNG) that removes misincorporated uracil in single-strand DNA in front of cytosine and adenine substrates (Peña-Diaz *et al.*, 2004). Evidence of a canonical BER pathway was provided by a study that reported that the function of TcUNG is increased *in vitro* in the presence of AP endonuclease (Farez-Vidal, 2001), suggesting that these two proteins act on the same pathway. The long-patch repair pathway also seems to be present in all forms of *T. cruzi* since a recent paper described the presence of Tc-Flap endonuclease 1 (TcFEN1), a homolog of human FEN1. In this work, TcFEN1 increased parasite resistance to oxidative stress when overexpressed; this protein deals with DNA intermediates containing a 5’ flap, showing a protein specific to LP-BER in this parasite (Ponce *et al.*, 2017).

Among DNA modifications caused by oxidative stress, the most prevalent is the generation of 8-oxodG. Nevertheless, several groups of organisms have a BER subpathway, the GO system, which can handle damage caused by 8-oxodG (Michaels and Miller, 1992; Nash *et al.*, 1996; Slupska *et al.*, 1996; Radicella *et al.*, 1997; Kuipers *et al.*, 2000; Barzilai and Yamamoto, 2004; Hwang *et al.*, 2014; Boiteux *et al.*, 2017). Three proteins from the GO system are significant in prokaryotic cells: MutM (also named FPG), repairs 8-oxodG already misincorporated to cytosine. MutY, removes an adenine misincorporated in front of 8-oxodG in double-stranded DNA, and MutT hydrolyzes 8-oxod-GTP into its monophosphate form, preventing its misincorporation into the DNA molecule by DNA polymerase (Michaels and Miller, 1992).

For *T. cruzi*, all three proteins of the GO system have been characterized (Furtado *et al.*, 2012; Aguiar *et al.*, 2013; Kunrath-Lima *et al.*, 2017). Initially, only TcOGG1 (FPG homolog for *T. cruzi*) and TcMYH (MutY homolog for *T. cruzi*) were identified as part of the *T. cruzi* CL Brener strain genome deposited in TritrypDB (El-Sayed *et al.*, 2005). Heterogeneous expression of TcOGG1 in *Ogg1* mutants of *Saccharomyces cerevisiae* could complement the deficient phenotype of the yeast. In *T. cruzi*, *OGG1* is located on nuclear and kinetoplast DNA. The protein was also functional in both organelles as overexpression sensitized the parasite to oxidative stress and led to lower rates of 8-oxodG (Furtado *et al.*, 2012). A similar result was observed for TcMYH, which is found in nuclear and kinetoplast DNA; overexpression of this protein led to increased sensitivity of the modified strain (Kunrath-Lima *et al.*, 2017). In addition, the parasite possesses a functional *MutT* homolog, which can complement *MutT*-deficient *E. coli*; overexpression of this protein increased resistance, decreasing parasite DNA damage (Aguiar *et al.*, 2013). The difference observed after overexpression of each protein can be explained by their function. MutY and OGG1 are proteins that act directly on the lesion already incorporated into the DNA strand, generating AP sites for the BER pathway to repair; however, MutT acts before incorporation, by removing the modified base as a substrate for the DNA polymerase (Michaels and Miller, 1992) and their homologs may act in the same way in *T. cruzi*. Thus, TcMYH and TcOGG1 overexpression may cause an imbalance in the DNA repair system of these modified strains, generating more AP sites than the parasite has the ability to efficiently repair. In fact, strains overexpressing TcMYH accumulate more AP sites after treatment with H_2_O_2_ and have a higher and earlier peak of AP sites in the genome as compared to the WT strain (Kunrath-Lima *et al.*, 2017). By using these mutants it was demonstrated that overexpression of TcMTH, but not TcMYH or TcOGG1, leads to higher resistance to BZN (Rajão *et al.*, 2014). This result indicates that BZN has a preference for action on the nucleotide pool and also targets parasite DNA; thus this is a direct link between DNA metabolism and Chagas disease treatment.

Regarding infection capability, overexpression of TcMTH increased both parasitemia and the number of amastigotes per cell *in vitro* as compared to the WT strain (Aguiar *et al.*, 2013; Goes *et al.*, 2016). An intriguing finding was that, after pre-treatment with a sublethal dose of H_2_O_2_, the number of intracellular parasites per cell after infection was higher when the authors compared the non-treated overexpressing cells with the pre-treated overexpressing cells (Aguiar *et al.*, 2013). Also, when an experiment was performed using Phox KO macrophage (i.e., mice deficient in the gp91^phox^ subunit of the NADPH oxidase), which impairs ROS production post-infection, the authors found that the parasitemia in modified macrophages was lower than in control cells (Goes *et al.*, 2016). These results are in agreement with previous works that had described oxidative stress as a factor that enhances the parasite infection (Paiva *et al.*, 2012; Paiva and Bozza, 2014), but the exact signal that these ROS give remains unclear.

The investigation into the importance of the response to ROS for *T. cruzi* was based on the finding that the parasite lacks, in its annotated genome, the sequence for the catalase gene (Boveris *et al.*, 1980; El-Sayed *et al.*, 2005; Freire *et al.*, 2017). Catalase is an antioxidant enzyme found in nearly all aerobic organisms. It performs the decomposition of hydrogen peroxide into water and oxygen (Chelikani *et al.*, 2004), two molecules that are not harmful to most organisms. The lack of such an essential gene was unexpected given the importance for *T. cruzi* to fight oxidative stress, and given the central role catalase plays in oxidative defense in several organisms (Chelikani *et al.*, 2004). The most exciting finding was that the heterologous expression of the catalase gene from *E. coli* (*KatE*) in *T. cruzi* increased parasite resistance to hydrogen peroxide. However, when the cells were pre-treated with a sub-lethal dose of H_2_O_2_, there was no difference in survival as compared with *KatE* in WT strains (Freire *et al.*, 2017). These results suggest that the pre-treatment can induce a cellular adaptation in WT strains of *T. cruzi*, a condition that is abrogated with the expression of the heterologous *KatE* gene. *T. cruzi* modified with *KatE* also exhibited higher parasitemia, infection index, and midgut proliferation in the invertebrate host when compared to WT cells (Freire *et al.*, 2017). Taken together, these results help illustrate a scenario in which ROS provides a signal for *T. cruzi* proliferation in cells, as observed in other works (Paiva *et al.*, 2012) and that, the abolishment of this signal caused by KatE, which degrades H_2_O_2_ into two non-signaling molecules, can alter the parasitic response to this stress and allow *T. cruzi* to benefit from oxidative stress to replicate in their hosts.

**Figure 1:**
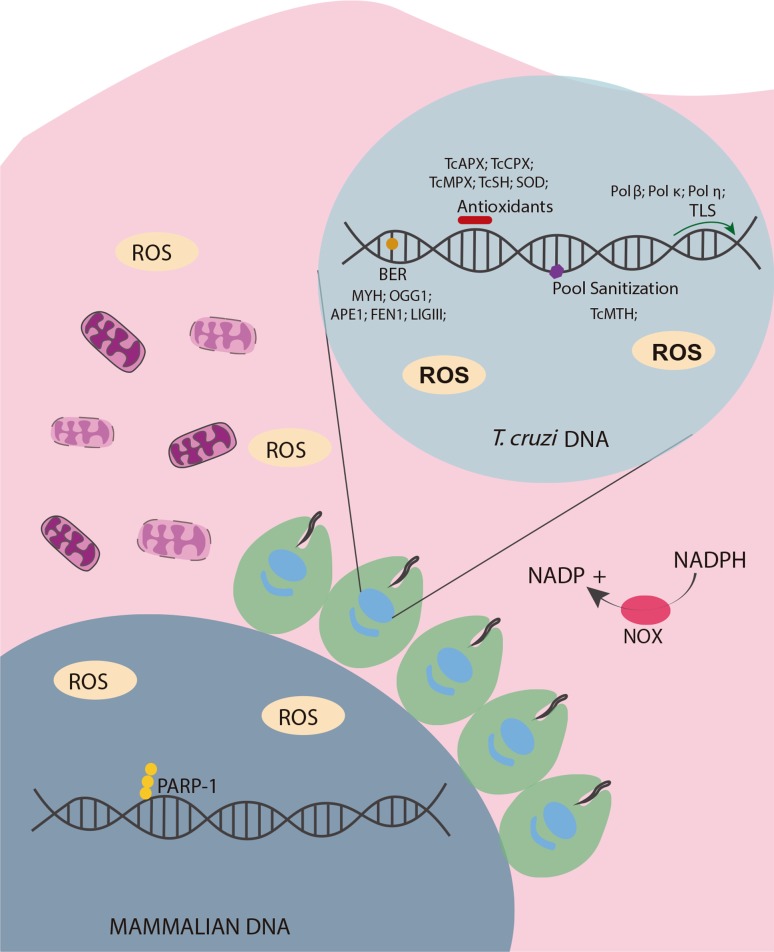
Oxidative stress in parasite-host interaction in *T. cruzi.* After infection, the parasite faces oxidative stress induced by the host cell. Parasites have several defense mechanisms to prevent damage to the nuclear and kinetoplast DNA. The first line of defense is composed of several antioxidants proteins; of these, TcAPX, TcCPX, TcMPX, TcSH, and SOD proteins are the best characterized. ROS that escape from the antioxidant system can cause DNA damage when they encounter parasite DNA molecules (8-oxoG is the most common modification that arises from this stress). To prevent the deleterious effects, the parasite has a specific BER subsystem (GO System), that directly interacts with the modified base within the genome (TcOGG1) or any mispair with this base (TcMYH). The BER repair system has been extensively studied in the parasite and proteins from all steps of this repair pathway have been characterized. The last component of the GO system, the TcMTH protein, has also been identified in *T. cruzi*. If any damage persists in the parasite’s nuclear or mitochondrial genome, translesion polymerases have also been identified, demonstrating that the parasite has means to survive massive amounts of oxidative stress.

The parasite response to the insults caused by the host cell is, therefore, very complex and is summarized in Figure 1. Oxidative stress is related to several kinds of damage (Imlay, 2013), but the investigation into the relevant parasitic machinery to combat the resulting DNA damage demonstrates that repair and signaling machinery is essential for the parasite to sustain infection both *in vitro* and *in vivo*. However, the exact role of this signal in amastigotes is not yet clear. The majority of experiments were performed on the epimastigote and trypomastigote forms of the parasite, and investigation of the particularities of the amastigote form remains an interesting field of study.

## Mitochondrial DNA damage response

Although the response to oxidative stress is a significant area of research in *T. cruzi,* since the oxidative burst is the major insult the parasite faces during infection, little is known regarding parasitic mitochondrial ROS generation and mitochondrial DNA repair. It has long been reported that some DNA repair pathways are absent from the mitochondria. For over thirty years, there have been reports of BER proteins in the mitochondria (Anderson and Friedberg, 1980; Pettepher *et al.*, 1991). Repair of double-strand breaks has already been described in some organisms such as fungi and plants (Manchekar *et al.*, 2006; Morel *et al.*, 2008; Mileshina *et al.*, 2011), but mammalian cells possess only a basic recombination machinery in the mitochondria (Thyagarajan *et al.*, 1996). Mitochondrial recombination events are rare, and some of the necessary components are involved in other DNA metabolic functions (Minczuk *et al.*, 2011). Mismatch resolution in mitochondrial DNA has only been recently described in some human cell lines and some other mammals (Mason *et al.*, 2003; Souza-Pinto *et al.*, 2009). Interestingly, general MMR nuclear proteins are not located in mitochondria, suggesting that different pathways occur in this organelle (Kazak *et al.*, 2012). Further details about specific pathways present in mitochondria of higher eukaryotes have already been reviewed in other works (Shokolenko *et al.*, 2011; Kazak *et al.*, 2012; Alexeyev *et al.*, 2013). *T. cruzi* and other trypanosomatids possess a single and modified mitochondria, allowing their use as great model organisms to study mitochondrial DNA repair. *T. cruzi* has been reported to be able to repair both the nuclear and mitochondrial DNA damaged by oxidative stress, although the level of kDNA damage is lower and more consistent than in the nuclear DNA. In addition, DNA repair in the kinetoplast does not eliminate all damage, as detected by a qPCR assay (Furtado *et al.*, 2012).

The main type of DNA damage generated by oxidative stress is the 8-oxodG base modification, which can be repaired by the GO system. In the *T. cruzi* GO system, two proteins colocalize with kDNA. Although TcOGG1 is generally thought to be localized within the nucleus, it was also identified in the kDNA *T. cruzi.* In addition, mitochondrial 8-oxodG levels were reduced in TcOGG1 overexpressing strains when compared to the control strain (Furtado *et al.*, 2012). TcMYH was also co-localized in the kinetoplast of *T. cruzi*, although the localization demonstrated that the protein was also found in the parasite’s nucleus. Overexpression of this protein caused a small elevation in the number mtDNA damage sites, as measured in mitochondria 30 minutes after the damage was induced and sensitized the cells to treatment with a specific mitochondrial stress agent (Kunrath-Lima *et al.*, 2017).

During the resolution of oxidative damage by BER, one pathway involves the insertion of the correct nucleotide into the 3’-OH of the AP site previously generated, followed by the excision of the remaining sugar-phosphate bone (DRP-lyase activity), a process that can be catalyzed by DNA polymerase β (Pol β) in some organisms (Allinson *et al.*, 2001). In *T. cruzi,* Pol β is present and is also able to perform these activities (Lopes *et al.*, 2008; Schamber-Reis *et al.*, 2012). It was demonstrated that this protein can deal with oxidative lesions in the kinetoplast and that its localization is dependent on the cell cycle. During oxidative stress, the protein translocates into the antipodal sites of the kinetoplast (Schamber-Reis *et al.*, 2012). Pol β PAK, a homolog of Pol β that contains a domain rich in proline, alanine, and lysine, has also been characterized in *T. cruzi* (Lopes *et al.*, 2008). Pol β PAK has a strict localization in the kDNA of *T. cruzi* and also possesses DRP-lyase activity. However, unlike Pol β, only Pol β PAK is capable of performing a bypass of 8-oxodG (Lopes *et al.*, 2008).

DNA polymerase kappa was also implicated in kDNA metabolism in *T. cruzi* (Rajão *et al.*, 2009)*.* Although the parasite possesses more than one sequence of DNA polymerase kappa, one of the copies possesses a mitochondrial localization signal and specific kinetoplast localization. Like polymerase β and β-PAK, DNA polymerase kappa can bypass 8-oxodG damage, but can also replicate an intermediate recombination structure *in vitro* that mimics the D-loop (Rajão *et al.*, 2009). Overexpression of these three polymerases increases parasite survival against BZN (Rajão *et al.*, 2014), another direct link between DNA metabolism and Chagas disease treatment. It is worth noting that only DNA polymerase kappa (Rajão *et al.*, 2009), TcOGG1 (Furtado *et al.*, 2012), and TcMYH (Kunrath-Lima *et al.*, 2017) possess a predicted mitochondrial localization signal; also, some kinetoplast associated proteins have no mitochondrial localization prediction, even though they are located in the organelle (Souza *et al.*, 2010). Some other proteins, such as Rad51 can also be localized to the mitochondria, as seen by by immunofluorescence experiments, although they are also not predicted to be present in this organelle (Cerqueira *et al.*, 2017), suggesting that the exportation of proteins to the mitochondria of *T. cruzi* still needs further study.

These data contribute to a scenario in which *T. cruzi* possesses, on the kinetoplast, proteins from all steps of the BER pathway, suggesting that the parasite can repair oxidative damages on the kDNA. Although the decrease in the number of lesions in the mitochondrial DNA observed in WT cells by qPCR is not significant (Furtado *et al.*, 2012), the increase in the number of lesions on the kDNA caused by the overexpression of TcMYH (Kunrath-Lima *et al.*, 2017) demonstrates that the kinetoplast does possess proteins for combating oxidative damage within this organelle. The quantification of DNA damage from other sources and repaired by other DNA repair pathways remain to be investigated.

## Chronic Chagas disease and DNA metabolism

A remarkable characteristic of Chagas disease is the heterogeneity of its clinical manifestations. Immediately after the infection, patients initiate an acute phase of the disease that can last from 4-8 weeks and is, in most cases, asymptomatic (Prata, 2001; Pérez-Molina and Molina, 2018). Symptomatic acute phase patients can present symptoms like fever, inflammation at the location of infection, and unilateral palpebral edema (a clinical condition called Romaña sign). In severe cases, the acute phase can lead to acute myocarditis, meningoencephalitis, and pericardial effusion (Laranja *et al.*, 1956; Teixeira *et al.*, 2006; Pinto *et al.*, 2008; Pereira and Navarro, 2013). However, the disease usually persists in an asymptomatic form and enters a chronic stage, which is characterized by low parasitemia (Pereira and Navarro, 2013). Only 30% to 40% of these patients develop cardiomyopathy, megaesophagus, or megacolon (Prata, 2001; Rassi *et al.*, 2010; Pérez-Molina and Molina, 2018). It has been long known that treatment with BZN and nifurtimox (NX) provides a high cure rate when given during the acute phase (Bahia-Oliveira *et al.*, 2000), but this treatment is 4 to 16 times less effective during the chronic phase (Cançado, 2002). In this context, one of the biggest questions regarding Chagas disease is why the parasite can persist in such a long period of dormancy, in which the host exhibits low levels of parasitemia, lasting for years, and the parasite becomes resistant to treatment.

Recently, an interesting report revealed that during cellular infection the parasite develops a non-proliferating intracellular amastigote form (Sánchez-Valdéz *et al.*, 2018). By using a cell division tracker and the 5-ethynyl-2’-deoxyuridine (EdU) thymidine analog, the authors were able to demonstrate that, although the BZN treatment is effective in reducing the number of trypomastigotes during the acute phase, there are still some amastigotes in dormancy and in a non-replicative state. Remarkably, the authors observed that, *in vivo* and *in situ,* even a single dormant amastigote cell can resume infection after this latency period, where the cells are dormant and in a non-replicative state (Sánchez-Valdéz *et al.*, 2018). When those cells were challenged with BZN, those dormant amastigotes were resistant to treatment (Sánchez-Valdéz *et al.*, 2018). Although it is not clear if BZN causes this dormancy, or if the parasite is already dormant and the treatment is unable to affect it since, as stated previously, BZN causes oxidation mainly on the parasite nucleotide pool (Rajão *et al.*, 2014).

A state that resembles dormancy has already been previously reported for *T. cruzi* in epimastigote cultures under laboratory conditions. After exposure to high doses of gamma irradiation (i.e. 500 Gy), epimastigotes enter a non-replicative state for a period of time that could be as long as 10 days (Regis-da-Silva *et al.*, 2006; Alves *et al.*, 2018; Silva *et al.*, 2018), although parasite DNA is repaired during the first 48 hours after the damaging insult (Regis-da-Silva *et al.*, 2006). This growth impairment was coincident with an arrest in S/G2 phase, which was diminished by the time of growth resumption (Garcia *et al.*, 2016), indicating that those cells were not replicating. It has also been shown that resumption of parasite DNA repair and growth are dependent on Rad51 levels (Regis-da-Silva *et al.*, 2006; Silva *et al.*, 2018). Rad51 seems to be related to specific kinds of replicative stress, being necessary for the response to methyl methanesulfonate (MMS), an alkylating agent capable of causing stalled replication forks; however, RAD51 is not recruited in response to replicative stress caused by hydroxyurea (Silva *et al.*, 2018). The relationship between those two dormant states needs to be investigated as it is not yet clear if they represent correlated events. Therefore, the control of replication and dormancy could be essential for *T. cruzi* survival.

It has recently been reported that in Chagas disease, dormancy is a mechanism by which *T. cruzi* can resist treatment with BZN, as demonstrated in both during *in vivo* and *in vitro* systems (Sánchez-Valdéz *et al.*, 2018). This dormancy can be replicated in the laboratory by causing DNA damage and replication stress (Regis-da-Silva *et al.*, 2006; Cerqueira *et al.*, 2017; Alves *et al.*, 2018; Silva *et al.*, 2018). Furthermore, it was recently demonstrated that different *T. cruzi* discrete typing units (DTUs), clusters of parasites based on biogeographical data, sequencing, and similarity among strains, exchange genetic information (Alves *et al.*, 2018), but the relationship between these phenomena and the differential virulence observed among these clusters has not yet been explored.

As with the discovery of the mechanism of BZN resistance (Rajão *et al.*, 2014), researchers have demonstrated a direct interplay between DNA metabolism and repair during Chagas disease development. It will be of interest to determine how the DNA damage response can alter the parasite metabolism in a broader sense. Although the kinetics of some DNA repair pathways were previously elucidated, much more investigation is needed on the sensing and signaling to the oxidative environment and their related responses.

## Effects of oxidative stress induced by *T. cruzi* in the host cell

The intracellular component of the *T. cruzi* life cycle begins with metacyclic or bloodstream trypomastigotes actively invading a host cell by disrupting the host cell membrane, stimulating cytosolic Ca^2+^ influx (Moreno, 2004; Tardieux, 2004). This event leads to several changes in the host cell, including mitochondrial membrane permeabilization and the induction of high levels of cytosolic ROS, which can signal apoptosis (Kroemer *et al.*, 2007; Giorgi *et al.*, 2012; Webster, 2012). The imbalance in ROS and antioxidant barriers has been described in many pathogen infections (Piacenza *et al.*, 2019). During *T. cruzi* infection, NADP oxidase (NOX) 2 was reported to be present in the plasma membrane of macrophages (Cardoni *et al.*, 1997). In addition, high levels of superoxide (O_2_^-^) and nitric oxide were released approximately 60-90 min after infection (Muñoz-Fernández *et al.*, 1992; Alvarez *et al.*, 2004). ROS generated in the intraphagosomal space is likely to have microbicidal effects on *T. cruzi*, which were reversed when *T. cruzi* cytosolic tryparedoxin was overexpressed. Higher levels of parasite survival after infection were observed. In addition, membrane potential loss from host cell mitochondria was observed after parasite cardiomyocyte infection. At 48 h post-infection, increased levels of cytosolic O_2_^-^ were observed, even when ROS producing enzymes from host cells were pharmacologically inhibited (Gupta *et al.*, 2009). The relationships between infection and oxidative stress are reviewed elsewhere (Gupta *et al.*, 2011; Paiva *et al.*, 2018).

As mentioned above, ROS produced by the host cell have a microbicidal effect on *T. cruzi* in the host cell environment (Muñoz-Fernandez *et al.*, 1992; Alvarez *et al.*, 2004). Recently, it was proposed that *T. cruzi* utilize their response to oxidative stress to survive inside the host cell cytosol. Briefly, macrophages and mice treated with cobalt protoporphyrin (CoPP), an antioxidant that induces heme oxygenase (HO-1) expression through nuclear erythroid factor-2 (NRF-2), reduced parasite growth. Paiva *et al.* (2012) observed that the treatment of infected cells with several antioxidants (apocynin, superoxide dismutase, N-acetyl-L-cysteine, resveratrol) reduces parasite burden. Also, there is evidence that BZN, the drug used to treat Chagas disease, stimulates NRF2 (Rigalli *et al.*, 2016). Conversely, when they treated infected cells with a respiratory burst inducer, phorbol 12-myristate 13-acetate (PMA) and H_2_O_2_, parasite burden increased significantly. These results raise an important question: could oxidative stress not only benefit the parasite but also prejudice host cell? Thus, how the oxidative stress induced by *T. cruzi* could harm the host cell is not yet fully understood.

There are some studies reporting that *T. cruzi* can interfere with the host cell cycle, although the mechanisms behind this process have not yet been fully described. The transcriptome revealed downregulation of cell cycle and cell division genes (Shigihara *et al.*, 2008; Costales *et al.*, 2009), though there was no difference in S-phase between uninfected cells and infected cells after 48 h of infection (Costales *et al.*, 2009). Our recent findings suggest that infected cells are less likely to be in S phase as compared to control.

Several genes related to DNA metabolism are up-regulated during early timepoints of infection (3 h and 6 h post-infection), such as the DNA polymerases *POLK* and *POLB*, DNA helicase *RECQL*, and DNA glycosylase *SMUG1,* that are involved in various types of DNA repair, including mismatch repair, base excision repair, and direct repair (Costales *et al.*, 2009). It was also reported that *T. cruzi* induces DNA breaks in cardiomyocytes during late stages of infection; the breaks are repaired when cells are treated with a free radical ion trap (PBN). PAR levels were increased in *T. cruzi* infected cells (Ba *et al.*, 2010). These results suggest that oxidative stress leads to BER pathway activation in the host cell during infection. The mechanisms behind genotoxicity in host cells are not yet fully understood and research efforts toward this end are underway.

There is some evidence that *T. cruzi* infection can induce not only host cell cycle arrest, but might also lead to senescence (Guimarães-Pinto *et al.*, 2018). It was revealed that a senescence-associated secretory phenotype (SASP) characterized by the production of a specific set of cytokines and chemokines such as IL-6, TNF-α, IL-1β, and MCP-1 and also secretion of SA-β-galactose are higher in infected fibroblast cells when compared to non-infected cells. The authors observed that host cell nuclei presented a characteristic senescence-associated heterochromatin focus. In addition, they observed that antioxidants reduced SASP molecules in the host cell and inhibited parasite growth. Cellular senescence, by definition, impairs cell cycle progression, which could benefit amastigote multiplication in the host environment. These results suggest that parasite growth in fibroblasts leads to host cell senescence, which benefits *T. cruzi* reproduction in the host cell cytosol.

In cell culture after parasite growth, the host cell will eventually die, spreading parasites to the supernatant. How the parasite induces host cell death is controversial. It has been reported that *T. cruzi* can induce apoptosis in the host cell (Stahl *et al.*, 2013). The authors observed activation of caspases 3/7, 8, and 9 in cardiomyocytes infected with *T. cruzi* when compared to control cells. In this model, STAT3 activation was induced by infection. Therefore, the Janus Kinase (JAK) signal-STAT pathway could be responsible for apoptosis and cardiomyopathy. Conversely, it was reported that *T. cruzi* could prevent cardiac cells from undergoing apoptosis via activation of NF-kB and IL-1 (Petersen *et al.*, 2006). How the parasite contributes to cell death and how the cell fate decision might spread neighboring cells is an important issue that needs to be further study.

## Concluding remarks

As described above, DNA metabolism and responses to DNA damage in both the parasite and host vary depending on the parasite in question and its specific life form. In Chagas disease, success of the infection and associated pathological effects are influenced by both parasite and host factors. (Andrade *et al.*, 1999; Andrade and Andrews, 2005; Albertti *et al.*, 2010; Kayama and Takeda 2010; Henrique *et al.*, 2016). Parasite-host interactions have been studied for a long time and seem to be important for both cell types (Caradonna and Burleigh, 2011; Caradonna *et al.*, 2013). The findings that *T. cruzi* can alter the mitochondrial membrane potential and the oxidative burst within the host cell to their benefit gives a new perspective on the infection process. Investigation into how the parasite regulates both its own and the host cell cycle is also essential, as this information could lead to new insights into the understanding of parasite/host interactions. Reports of how *T. cruzi* can regulate host cell cycle arrest and/or cell death in cardiomyocytes and further understanding of how the parasite enters a dormant state can lead to a new understanding of the disease mechanisms.
